# High Accordance in Prognosis Prediction of Colorectal Cancer across Independent Datasets by Multi-Gene Module Expression Profiles

**DOI:** 10.1371/journal.pone.0033653

**Published:** 2012-03-16

**Authors:** Wenting Li, Rui Wang, Zhangming Yan, Linfu Bai, Zhirong Sun

**Affiliations:** 1 Ministry of Education Key Laboratory of Bioinformatics, State Key Laboratory of Biomembrane and Membrane Biotechnology, Institute of Bioinformatics and Systems Biology, School of Life Sciences, Tsinghua University, Beijing, People's Republic of China; 2 Computational Biology and Bioinformatics Program, Institute for Genome Science and Policy, Duke University Medical Center, Durham, North Carolina, United States of America; University of Texas MD Anderson Cancer Center, United States of America

## Abstract

A considerable portion of patients with colorectal cancer have a high risk of disease recurrence after surgery. These patients can be identified by analyzing the expression profiles of signature genes in tumors. But there is no consensus on which genes should be used and the performance of specific set of signature genes varies greatly with different datasets, impeding their implementation in the routine clinical application. Instead of using individual genes, here we identified functional multi-gene modules with significant expression changes between recurrent and recurrence-free tumors, used them as the signatures for predicting colorectal cancer recurrence in multiple datasets that were collected independently and profiled on different microarray platforms. The multi-gene modules we identified have a significant enrichment of known genes and biological processes relevant to cancer development, including genes from the chemokine pathway. Most strikingly, they recruited a significant enrichment of somatic mutations found in colorectal cancer. These results confirmed the functional relevance of these modules for colorectal cancer development. Further, these functional modules from different datasets overlapped significantly. Finally, we demonstrated that, leveraging above information of these modules, our module based classifier avoided arbitrary fitting the classifier function and screening the signatures using the training data, and achieved more consistency in prognosis prediction across three independent datasets, which holds even using very small training sets of tumors.

## Introduction

Colorectal cancer is one leading cause of cancer mortality. About 20–30% of patients at stage II and 50% of patients at stage III experience disease recurrence after surgery [1]. Accuracy and stability of the prognosis prediction are critical when determining the appropriate therapy scheme regarding different recurrence risk. The recent studies have suggested the expression profile of multi-gene signatures as a better prognosis predictor for patients with colorectal cancer than traditional methods using clinical or pathological features, and some are entering the market [2]–[7]. These signature genes were typically identified from differentially expressed genes between a training set of tumors from patients with or without disease recurrence. Their expression data were then used to train a statistical classifier that can best discriminate the two groups of training tumors. In some cases, these steps, i.e. the gene selection and classifier construction, are iterated to optimize both choices.

One major problem with these multi-gene classifiers is that their signature genes vary significantly for different cohorts of studies, different populations of patients, and different microarray platforms, presumably due to the low accordance between microarray expression data [8]. To get a consensus list of signature genes, it is estimated that thousands of tumor samples would be needed for training such classifiers [9]. As a result, the several reported sets of signature genes highly depended on the training samples and had only overlap minimally [10]. Another concern is that the choice of a statistical classifier is arbitrary and lacks explicit biological basis, so that the classifier may be over-fitted by the dataset from which it was invented. For example, in one recent study, the multi-gene classifiers constructed from one dataset were cross-validated in a different dataset to find that their prediction accuracy was substantially reduced [3]. Such reduction was due to some missing genes in the gene classifier relative to the best classifier constructed from the cross-validation dataset. Therefore, these factors have led to high variability in the predictive performance of multi-gene classifiers and limited their generalized usage in clinical practice.

Recently, higher accordance across different microarray dataset has been reported in the expression patterns of multi-gene modules, i.e. groups of functionally related genes [11]–[14]. Motivated by this finding, we aimed to identify such modules by combining both gene expression and protein interaction data and used the most differentially expressed modules to construct a novel classifier. Importantly, we verified that these modules are non-randomly associated with colorectal cancer recurrence in different datasets, and that the modules from different datasets overlap by significantly more genes than random, indicating the overlapping percentage of the top ranked modules possessed discriminative power. In this way, we avoided the use of the low-accordance gene signatures and an arbitrary statistical function to fit. We demonstrated its application to three independent datasets of colorectal cancer patients that profiled on different microarray platform and obtained reproducible predictions with accuracies of 74%, 76% and 68%, and AUC (area under ROC) values of 79%, 79% and 72% by Leave-One-Out validation. Reasonable accuracies are seen when decreasing the size of training sets (34, 10 or 18 tumors) and the variability across datasets remains low, which is ∼1/2 of existing multi-gene based classifiers.

## Materials and Methods

### Data source

#### Tumor expression data and preprocessing

Three public pre-processed microarray datasets of colorectal tumors as below were used; note that the classification of the patients, recurrent or non-recurrent, is referred to the actual status described in the original papers or description files:

1. German dataset [3]: It included 55 German patients with primary colorectal cancer (stage I and II), where 29 patients are disease recurrence free and their follow-up time at least 5.3 years after surgery. The expression of tumor samples was profiled on the Affymetrix HG-U133A platform.
2. Barrier dataset [5]: It included 50 patients with stage II colorectal cancer. 25 of them are disease recurrence free and their follow-up time at least 5 years after surgery. The expression of tumor samples was profiled on the Affymetrix HG-U133A platform.
3. GSE5206 [15]: It included 100 patients with stage I–IV colorectal cancer. 23 of them had disease recurrence after surgery. There is no information about their follow up time. Here we removed 37 samples with higher stage (III and IV) from the recurrent-free sets and leaved 63 patients for prediction validation. The expression of tumor samples was profiled on the Affymetrix HG-U133_plus_2 platform.

For each probe with missing values, we applied R package ‘impute’ [16] to fill with the average of its k-nearest neighbors Genes with multiple probes were processed by averaging their expression level.

#### Gene ontology data

Gene ontology (GO) data from the Molecular Signatures Database (MsigDB) v2.5 [17] were used, which included 1454 GO sets and 8299 genes.

#### Protein interaction data

The protein interaction data were downloaded from the HPRD database [18] (release 8) and BioGRID the database [19], which included 6511 nodes and 29694 interactions.

#### Known genes related with colorectal cancer recurrence

Colorectal cancer recurrence related genes were collected based on their annotations from two sources, respectively: OMIM database (www.ncbi.nlm.nih.gov/omim) [20] and online literature mining using PubGene (http://www.pubgene.org/) [21]. We obtained 41 related genes from OMIM database. Using PubGene, we first searched for genes associated with the term ‘colorectal cancer’ and ‘recurrence’ to obtain 2793 and 1609 genes, respectively, and then took the intersection of these two gene lists as the final set of 1038 colorectal cancer recurrence related genes.

#### Colorectal cancer somatic mutation data

The somatic mutation data for colorectal cancer is downloaded from COSMIC database [22] in the category of ‘the large intestine tissue’, not including the sub-tissue, anus and appendix, with all two histological terms: adenoma and carcinoma.

### Constructing GO co-expression networks

We built networks for each GO gene set. This was for three reasons: (1) it proved useful to incorporate prior information, e.g. genes within the same pathways, to facilitate computational methods in identification of functional modules [23]–[26]; (2) it allows multi-functional genes to be present in more than one functional modules; (3) many interaction data were obtained in-vitro and may not exist in physiological situations and therefore, limiting the interactions within a gene ontology may help reduce such false positives. In details, for each GO gene set, genes not present in the microarray dataset were removed. The remaining genes in each GO set are used as vertices of the network and the edges were drawn based on protein interaction data. Each vertex is associated with an ***n***-dimensional expression vector where ***n*** is the total number of tumor samples in the dataset. The value at each dimension is the expression level of this gene in the corresponding tumor sample. The edge between any two vertices is weighted by their co-expression level [27]. Here we chose the Pearson correlation coefficient to measure the co-expression level. Note that there are a few alternative metrics, e.g. Spearman correlation and mutual information, and these metrics generally led to similar results in network properties and module discovery [28]. Furthermore, Pearson correlation coefficient has been widely used and suggested to be a good way to handle noises within the microarray data [29], [30], since it measures the collaborative degree of two expression vectors but not the strength of them. Specifically, the weight of an edge between two vertices *i* and *j* is defined as the absolute value of person correlation coefficient between their expression vectors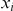, 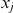:
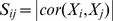
(1)


### Identifying functional modules

There are several methods to identify modular structures within a network and the choice of method varies with several factors, e.g. the network structures [31]. Considering the dense structure of each GO network, we applied the weighted Girvan and Newman (GN) algorithm [32] for module discovery. Compared to other existing methods that start with seed nodes and explore the vicinity for high scored modular structures [11], [33]–[36], the GN algorithm is edge-oriented and search for globally optimal modules. It is based on shortest-path algorithm, calculates the betweenness of all edges and repeated removes the edge with highest betweenness. Here, the betweenness score of an edge is defined by the sum of the all shortest paths passing through it and divided by its weight of corresponding edge. The original GN algorithm always cuts the dendrogram at highest Q value, which results in a large variation in the module size and sometimes huge modules with low biological coherence [37]. To avoid this problem, we required each module to contain no more than 20 genes. The detailed procedures are as follows:

1. Calculate betweenness scores of all edges in each GO network.
2. Find edge with the highest score and remove it from the graph.
3. Repeat above steps until no isolated graphs contain over 20 genes.
4. Singletons with only one gene were ignored.

### Rank differentially expressed modules between tumors with and without recurrence

The expression changes between tumors with and without recurrence were evaluated by our P-SAGE algorithm [38]. For a module ***s*** with a total of ***k*** genes, the score of differential significance (SDS) is defined by:
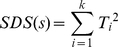
(2)where 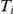 is the ***t*** score for ***i*** **-**th gene in the module ***s***. Noticing that the SDS scores correlates with the module size ***k***, we obtained their corresponding p-values from the chi square distribution 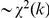, which are used to sort the identified functional modules in ascending. Modules with higher rankings, i.e. the most differentially expressed modules with smaller p-values, are used for evaluation and prognosis prediction.

### The prognosis prediction paradigm

#### The scheme of the prediction paradigm

Given a training set of tumor samples, we split it into two halves, [R1] and [R2], each with n non-recurrent and n-1 recurrent tumors. These two halves are considered as two independent datasets. Then, we assume the test tumor (i.e. unlabeled) X as recurrent and put it into [R1] and [R2], i.e. [R1+X] and [R2+X]. We identified the top N modules from [R1+X] and [R2+X], respectively, and if the test tumor X is associated with high risk of recurrence, the two sets of resultant modules should overlap substantially. We calculated the overlapping percentage (OPN) which is calculated by the ratios of their intersection and their union, after being normalized against the overlapping percentage of corresponding modules identified from [R1] and [R2]. To avoid potential bias with a specific split, we repeated random split and above for 10 times to obtain an average <OPN>. Finally, we computed <OPN> for different N = 100, 200… 500 and use the average as the predictive score <OP>. Higher <OP> score indicates a higher risk of recurrence associated with the test tumor X. In this way, we avoid the common strategy of optimizing an arbitrary kernel function that has no clear biological basis.

#### Evaluation and comparison

For each dataset, its tumor samples were divided into a training set and a test set. We reported the performance measure, accuracy and AUC, with R package, ROCR. In leave one out validation, one tumor was randomly chosen as the test set and the rest tumors are used as the training set. In this way, the prediction was conducted for n times, where n is the total number of tumors in the dataset. In validations with the number of training samples being 34, 18 or 10, we conducted the prediction for (n-34), (n-18) or (n-10) times. Then we randomly chose the training set of tumors for 5 times and reported the average, maximal and minimal performance. The performance was compared with other methods using these three microarray datasets.

## Results

We used two independent datasets of early colorectal cancer patients to verify the two key hypotheses: (1) the most differentially expressed modules are non-randomly associated with tumor recurrence; (2) such modules identified from different datasets will overlap significantly in more genes than random.

### Overview of most differentially expressed modules identification

**Figure 1:**
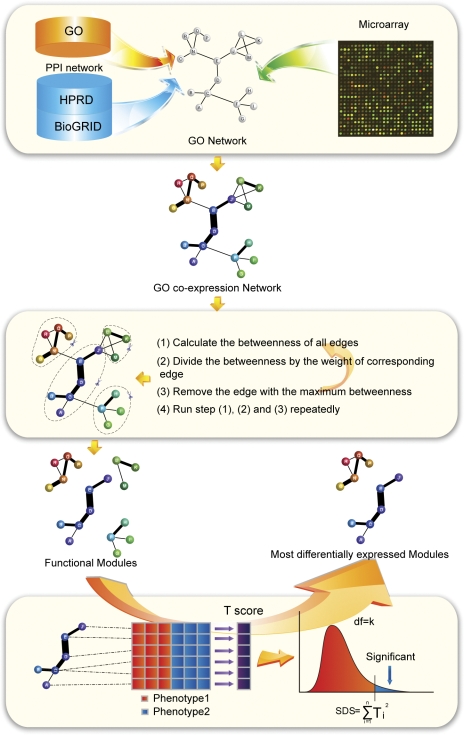
Schematic overview of most differentially expressed modules identification. Identifying the most differentially expressed modules include three key steps. First, the GO co-expressed network is constructed by combined the protein-protein interaction network, which was from the HPRD and BioGRID database, and GO gene sets together. The edges of network were weighed by co-expression level between their corresponding linked nodes. Second, functional modules were identified by the weighted Girvan-Newman algorithm [32]. Finally, functional modules were ranked on their differential levels between recurrent and non-recurrent tumors which were evaluated by the p-SAGE algorithm [38].

The identification of most differentially expressed modules included three key steps: network construction, topological module discovery, evaluation of differential expression at module level (Figure 1, more detailed description in METHOD AND MATRIERAL section). Briefly, we firstly clustered genes into large groups based on their GO annotation. As a gene may have more than one functional role, these GO groups may overlap in certain genes. Instead of constructing a single giant network, we used protein interaction data to build networks for each of these GO set of genes and identified multi-genes modules, i.e. groups of genes that are densely connected in network topology and relatively separate from the rest network. Lastly, the differential expression of each module between tumors with and without disease recurrence was ranked to obtain the top N modules for subsequent analysis.

The constructed GO networks contain 4428 genes in total for both Barrier and German datasets as they used the same microarray platform. We took the top 100, 200, …, 500 modules for subsequent analysis (Table S1). These modules have a differentially expressed p-value no greater than 0.005 in both German dataset and Barrier dataset.

### The most differentially expressed modules are non-randomly associated with tumor recurrence

**Figure 2:**
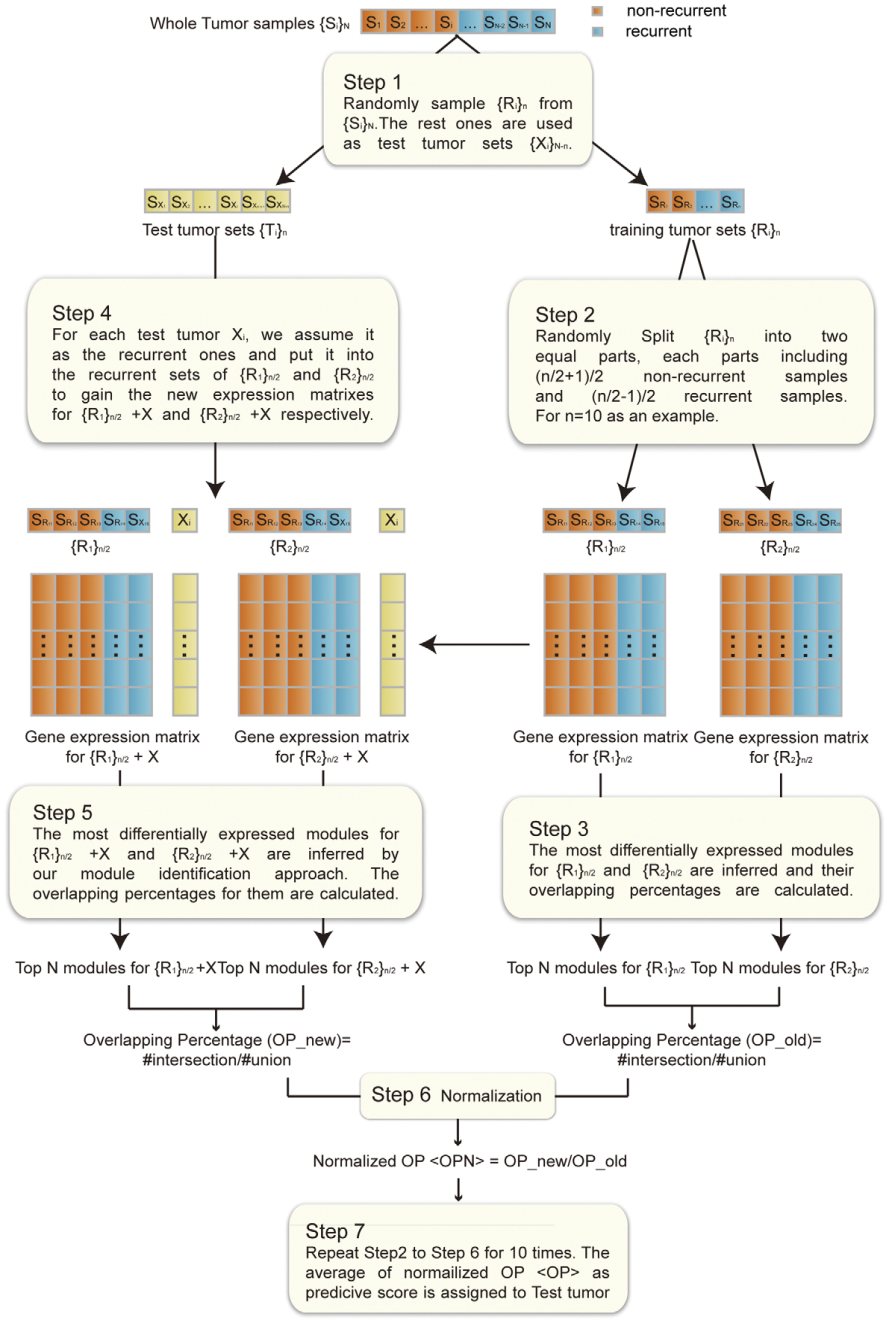
The percentage of known colorectal cancer (CRC) genes in top 50–500 MDMs inferred from German dataset. Known CRC genes were collected from the PubGene (A) or OMIM (B). The percentages were compared with those in top differentially expressed genes (t-test genes) with the same number of genes in top ranked N modules, or GO gene sets with the same amount of top ranked N modules.

As can be seen in Figure 2, we found a significant enrichment of genes related with colorectal cancer recurrence in these modules identified from German dataset according to both OMIM and PubGene annotations (see Methods). For control purposes, we generated sets of a same amount of genes that are identified as the most differentially expressed using the individual gene based t-test (“t-test genes”), or the most differentially expressed GO gene sets ranked by P-SAGE. Compared to these two controls, we found the higher proportions of colorectal cancer recurrence related genes were in the top 50–500 modules. They are about 1.9∼3.5 times (OMIM) and 2∼2.7 times (PubGene) higher versus top ranked individual genes, 2.6∼4.7 times (OMIM) and 1.7∼2.1(PubGene) times higher versus top ranked GO gene sets (Figure 2). Similar results were also seen for Barrier dataset (Figure S1).

Specifically, in analyzing the German dataset, we found three chemokines (CXCL9, CXCL10 and CXCL11) and their shared receptor CXCR3 in the top 10 modules. This is consistent with the recent finding that CXCR3 and another ligand CXCL10 promote invasion-related properties in colorectal cancer [39], [40]. To see if these results were reproducible, we randomly split German dataset into two halves, each being a smaller dataset with 14 or 15 non-recurrent tumors and 13 recurrent tumors, identified the top 100 modules and check if these chemokine related genes would show up. We performed such random splits for 1000 times and counted the frequencies of genes that appear at least once in both halves for top 100 modules. Also, considering hub genes that have more interacting partners would have a higher chance to show up in more modules, we normalized the frequency of each gene against its connectivity. We found the three chemokines: CXCL10, CXCL9 and CXCL11, yet not their receptor CXCR3, appear the most frequent (30.5%–44.1%) in all 1,000 splits. However, we performed the same analysis on Barrier dataset and did not found any of the three chemokines to show up in the top 100 modules in any random split. However, we found 19 and 18 of the member genes in the chemokine signaling pathway (190 genes in total) as curated at KEGG database showed up at least once in top 100 modules in German dataset and Barrier dataset, respectively (Table S2). They overlapped by 9 genes (STAT2, STAT3, LYN, MAPK1, FOXO3, NFKB1, GSK3B, PAK1 and PTK2B). These results indicate a possibility that the top modules were able to capture substantial changes (10%) in the chemokine signaling pathway associated with tumor recurrence, and are reproducible across different datasets. But it may be hard to further get down to specific genes in these modules to use as robust markers.

**Figure 3:**
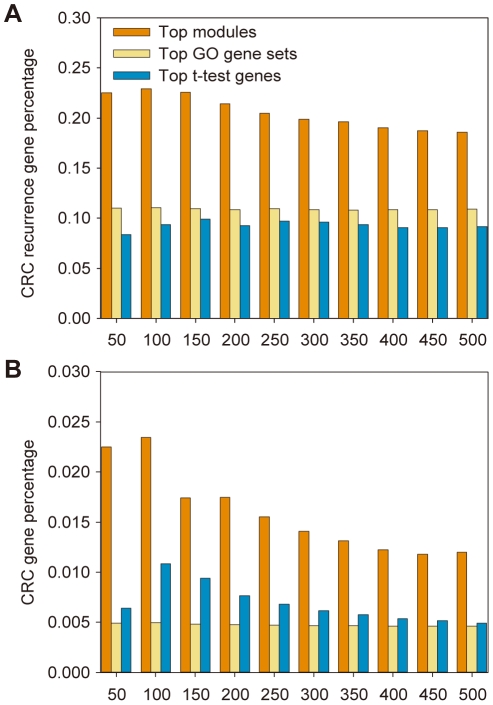
The enrichment levels of somatic mutations in top 50–500 modules inferred from the German datasets. By contrast, the controls are from the t-test gene and permutation test. T-test gene analysis was performed by using the same number of top differentially expressed genes as the number of genes covered by the corresponding top N modules.

As tumor develops with the accumulation of somatic mutations, we also assessed if there is a significant correlation between the top modules and the somatic mutations identified in colorectal cancer from COSMIC database. We first identified the modules that contain significant amount of mutations by Fisher exact test (p cutoff: 0.05). These modules were named as Mutated Modules (MMs). We then calculated percentages of MMs in top N modules and the rest modules to obtain an enrichment ratio. A higher ratio indicates a higher enrichment of mutations in the top N modules. For German dataset, we found its top 50–500 modules overlap significantly with MMs (Fisher exact test, p<0.002), with the enrichment scores around 3–4 (Figure 3). In contrast, we conducted a similar analysis on top genes of similar numbers identified by the conventional t-test (“t-test genes”) but found no significant overlap with genes in MMs (Fisher exact test, p-values>0.25). The percentages of mutated genes in top t-test genes vs. the rest genes are similar. To assess if the enrichment of mutations in top modules are associated with tumor recurrence, we permuted the labels of “recurrence” and “non-recurrence” to identify the top modules and found their enrichment ratios are about 1.3, which is comparable to those of the t-test genes. The similar results were also found in Barrier dataset (Figure S2).

To this end, we confirmed our first assumption that the identified top modules are non-randomly associated with tumor recurrence in two different independent datasets. Therefore, these modules may be used as more robust predictors than specific genes for prognosis prediction.

### The most differentially expressed modules had higher reproducibility

**Figure 4:**
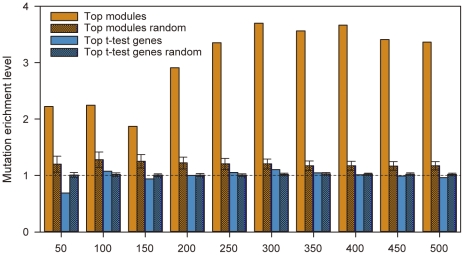
The percentage of overlapping genes in top 100–1000 modules identified from two independent datasets, German and Barrier. The overlapping percentage is calculated as the ratio for the number of intersection and union of the genes. We compared the percentage of overlapping genes on top ranked N modules, top t test genes with the same number of genes in top N modules, and their corresponding permutation test controls.

Next, we examined if the overlapping percentages of top modules are significantly higher than controls to be used as a discriminative metric. We identified top 100–1000 modules from Barrier and German datasets, respectively, and found these modules from the two different datasets overlapped significantly (p<1.75E-74). Their overlapping percentages (25.3%–54.9%) are over 7 times higher than the overlapping percentages of top t-test genes (3.3%–6.6%) and are also about 2 times of the mean overlapping percentages for top modules identified after permuting labels (Figure 4). Remarkably, these overlapping percentages are also higher than the extreme values obtained in the permutation cases, as outliers (Grubbs outlier test, p-values<0.006). Taken together, these results supported our second assumption and suggested the overlapping percentages of top modules are informative to predict tumor recurrence.

### A novel classifier based on the most differentially expressed modules can yield more robust prognosis predictions

**Figure 5:**
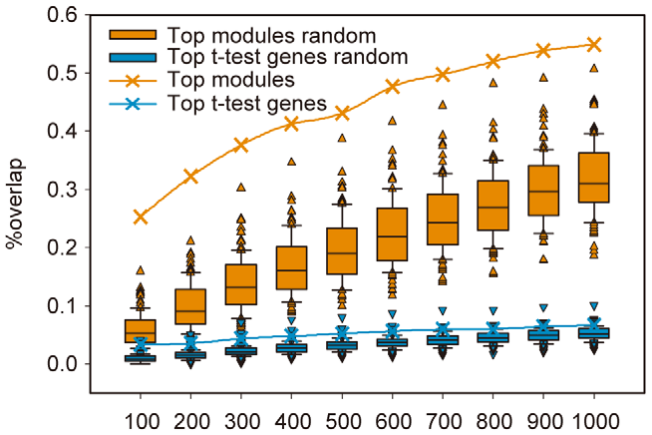
Schematic overview of classification and evaluation. The training tumor sets are first sampled randomly from the whole tumor datasets and then split randomly into two equal parts, each part including the non-recurrent and recurrent sets. Their corresponding top modules were inferred by the approach mentioned above and the overlapping percentage (OP_old) was calculated. For each test tumor X, we put it into the recurrent sets for both parts to constitute the new expression matrixes. The most differentially expressed modules for two new expression matrixes are inferred respectively. The overlapping percentage (OP_new) of these two sets of top modules is calculated and normalized by the OP_old. Considering the bias from the splitting at the step 2, the random splits were repeated for 10 times. The average of normalized OP is assigned to test tumor X.

Given above validations of our two key assumptions, we designed the prognosis prediction paradigm as follows. Briefly, we split the training set of tumors into two different sets. Each set contains both recurrent and non-recurrent tumors, so that the corresponding top modules can be inferred. An overlapping percentage (OP_old) of these modules from both sets was computed. Given a test tumor, we assumed it is “recurrent” and put it into each set to identify the new top modules and calculated the new overlapping percentage (OP_new). If the test tumor is “recurrent” as expected, the old and new overlapping percentages should be comparable; otherwise, the new overlapping percentages would be lower. In this way, we avoided using the specific genes but used the entire information of the top modules, since as shown above, only the latter is non-randomly associated with tumor recurrence. We also avoided the problematic step of fitting training tumor data to an arbitrary statistical function. Instead, the overlapping percentages of top modules were used which we showed should be of sufficient discriminative power. More details can be found in METHOD AND MATRIERAL section and Figure 5. In the following, we demonstrated the evaluation of this method in three independent datasets and compared its performance with that of previous methods using the same datasets.

#### Leave one out validation

**Figure 6:**
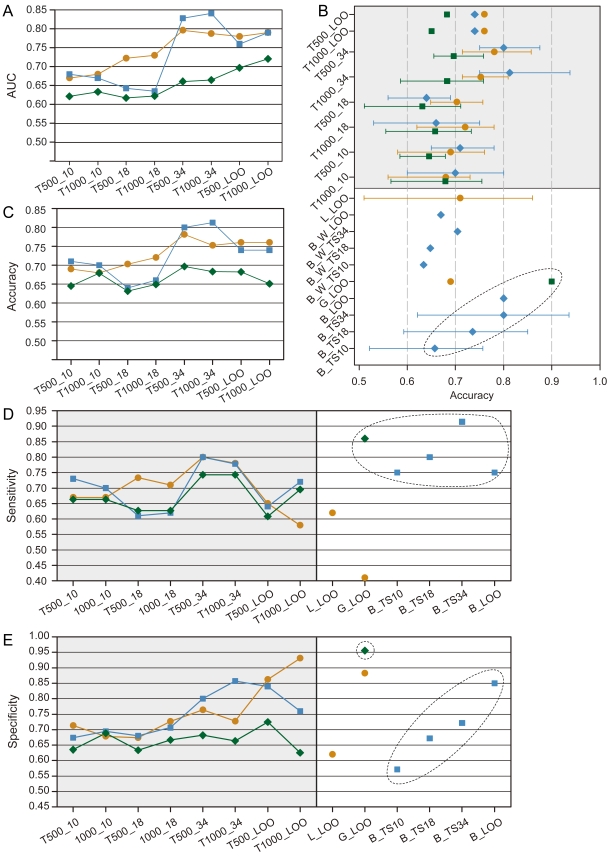
The prognosis prediction performance. The comparison of AUC (A) and accuracy (B) for three datasets: Different coloring schemes and shape indicate three independent datasets (orange circle: German dataset; blue diamond: Barrier dataset; green square: GSE5206 dataset). TX_Y methods (X: top 500 or 1000 MDMs; Y: 10 or 18 reference tumors or Leave-One-Out method (LOO)). The filled symbols denote the mean of AUCs; The comparison of accuracies(C), sensitivities (D) and specificities (E) for prognosis prediction between our method and present methods with same datasets, including the LOO results from Lin07 (L) [3], Garman08 (G) [42], Barrier06 (B) [5], and also the Barrier06's results obtained using 34 tumors (TS34), 18 tumors (TS18) or 10 tumors(TS 10) as the training set. The filled symbols are mean value. *The points in the dotted circle are the outcomes from the methods which were validated using makers discovered by the one and the same dataset.

We first evaluated the performance of our prediction method by Leave-One-Out validation, which is a popular choice used in previous studies. We reported the results of accuracy (the true positive rate at the point nearest to point (0,1) of the ROC), sensitivity, specificity and AUC to compare with existing multi-gene classifiers (Figure 6, the detailed information in Table S3). For German dataset, our method achieved higher performance than the recent two methods, an accuracy of 76%, about 5–7% higher (Lin07: 71%; Garman08: 69%), a sensitivity of 65%, about 3–24% higher (Lin07: 62%; Garman08: 41%), and a specificity of 93%, about 5–14% higher (Lin07: 79%; Garman08: 88%). For Barrier dataset, our method achieved an accuracy of 74%, a sensitivity of 72%, a specificity of 84%, which is slightly less than the Barrier06 results (accuracy: 80%; sensitivity: 75%; specificity: 85%) using this dataset and the resulting Barrier06 signatures. But it is much higher than another result using the same dataset and another Wang04 signature (accuracy: 67%). For GSE5206 dataset that has no specific follow up time, our method achieved the lowest but still reasonable accuracy (68%). It is also much lower than the accuracies achieved by the original methods invented using this dataset (90%; Garman08 method). However, we noted that this Garman08 method, when applied to a different dataset (German dataset), only achieved 69% accuracy. The about 21% difference of Garman08 method in different datasets may suggest a potential over-fitting problem of its classifier or an undesirably high variability in its performance. In contrast, our methods had much smaller variability (8% difference), with 74–76% accuracy for early stage (I or II) tumors in Barrier and German datasets, and 68% accuracy for stage I–IV tumors in GSE5206 dataset. The corresponding AUC values of our method were also similar across all three datasets: German - 79%, Barrier - 79% and GSE5206 - 70%.

To verify the samples size's impact on the prediction methods, smaller samples size at 34, 18, 10 have been carried out. The average value and the range (the minimum and maximum value) of accuracy, sensitivity, specificity and AUC are reported in each case (Figure 6, the detailed information in Table S3, and ROC curve in Figure S3).

#### Validation with 34 training samples

We randomly picked up n samples from each dataset, where n = 34, as training set to predict the recurrence risk for the rest tumors. For the German and Barrier datasets, the performances are much higher than the results in LOO validation. In detail, for German dataset, our method achieved an accuracy of 78%, AUC of 80%, a sensitivity of 80%, and a specificity of 76%. For Barrier datasets, it achieved higher accuracy of 81% and specificity of 86%, and less sensitivity of 78% than other methods (using Barrier signature: accuracy: 80%; sensitivity: 91%; specificity: 72%; using Wang04 signature: accuracy: 70%). In addition, our method only had much less variability (13% for Barrier dataset) than that of Barrier06 method (31%). For GSE5206 datasets, the performance is similar with the LOO validation, an accuracy of 70%, AUC of 66%, a sensitivity of 74% and a specificity of 68%.

#### Validation with 18 or 10 training samples

Next, we continued to decreased the size of the training set, n = 18 or 10, to validate the predict performance on the recurrence risk for the rest tumors.. In the case of n = 18, for German dataset, our method achieved an average accuracy of 72% for German dataset, better than all previous methods. For Barrier dataset, our method achieved an average accuracy of 66% and sensitivity of 63%, which are lower than the original Barrier06 signatures (74%), but similar as the Wang04 signatures (65%) and a higher specificity of 71% (Barrier06: 67%). We noted that, by sampling different sets of training tumors, our method had about half variability (13% for Barrier dataset) than that of Barrier06 method (26%). Lastly, our method had an average accuracy of 66% for the GSE5206 dataset, comparable to that of Barrier (72%) and German datasets (66%). The AUC quantities, sensitivity and specificity for all three datasets are consistent, too: accuracy: German - 73%, Barrier - 67% and GSE5206 - 75%; sensitivity: German – 71%, Barrier – 62%, and GSE5206 – 63%; specificity: German – 73%, Barrier – 71%, and GSE5206 – 67%.

In the case of n = 10, our method achieved an average accuracy of 69%, 71% and 74%, sensitivity of 67%, 73%, and 66%, specificity of 71%, 67%, 69% and the AUC of 68%, 68% and 63% for German, Barrier and GSE5206 datasets, respectively. Furthermore for Barrier datasets, our methods has a higher accuracy of 71% (using Barrier06 signatures: 66%, Wang04 signatures: 63%), and higher specificity of 67% (Barrier06: 57%).These results suggest our prediction can achieve highly reproducible and reasonable performance across different datasets, even with as few as 10 training tumors.

## Discussion

Tumor recurrence is associated with changes in different pathways. Such changes may be manifested in different genes for different patients. The existing signature genes were identified based on expression changes at individual gene levels, and thus may not necessarily capture such pathway level changes. Here we tackled this issue by constructing a classifier based on most differentially expressed multi-gene modules.

Many reported classifier with higher accuracy dependent tremendously on the training samples, indicated these classifier best fit the existing sample instead of the whole population of the interested phenotype. In this study we verified not only non-random biological qualitative association between top ranked modules with colorectal cancer recurrence in different datasets, but also higher quantitative overlapping measure of the modules from different datasets than random. Thus the overlapping percentage of the modules is used to be prognosis function to avoid arbitrary fitting by training data and potential over-fitting problem.

There are two things must be noted here. First, multi-gene modules or sets can be identified in a number of alternative ways, but they may not be informative enough to construct such a classifier. In one earlier study, modules associated with tumor metastasis of breast cancer were identified, but failed to find a significant enrichment of somatic mutations [11]. In some another studies, co-expressed gene set were found correlated with the tumor development but their size is too large [41]. Considering the definition of modules is still very liberal, we suggest that rigorous test on their biological relevance must be done before using them for constructing a classifier. Secondly, as shown in the case of chemokine pathway, it may be hard to further find robust signature genes to represent these signature modules. With only the expression data, the information of the modules needs to be exploited as much as possible. As a result, this leads to another critical feature of our classifier, using the overlapping percentages of modules as classifier function.

In conclusion we developed this novel module-based prognosis classifier to predict the outcome of patients with the colorectal cancer after surgery and have demonstrated that it yielded reasonable and reproducible performance across datasets with low variability. And it can also yield the satisfactory and commendable performance even at the case of fewer samples indicating it is cost-effective way which required only fewer amounts of tumor materials. The performance on the GSE5206 is relatively lower than other two datasets. No specific follow up time which may cause wrong classified label for the non-recurrent patients may be one of the reasons.

Furthermore compared with current prediction based on pathological staging, this prognosis classifier can help more to identify patients with higher recurrent risk and suggest better decisions of personalized treatment therapy. In future clinical application, the methods need to set several reference samples as the benchmark. For each test patient, we compared its gene expression profile with benchmark sets, and get the score based on our algorithm. The patients with higher overlapping score are the higher risk ones and should be received more intensive follow-up adjuvant therapy, whereas the patients with lower overlapping score are the lower risk ones and might be exonerated from the injury caused by more aggressive treatment. The module-based prognosis strategy also brings about a wider application in the other cancer types or other aspects, such as evaluating responsiveness of new drug or therapeutics.

For most existing methods, it requires huge number of samples to establish the diagnostic setting, which is usually costly and time consuming. However, with our method, only few samples are needed. Furthermore in our study, a wide range of the samples size (10, 18, 34, and n-1 (n: all samples), German: 55, Barrier: 50, GSE5206: 63) has been validated on our method, and yielded robust results to the sample size change, that is, our method's result will not be strongly affected by the sample size, thus gaining a unique advantage when applied to new region, new population and especially used in some new discover/rare cancers. Additional information, e.g. gene copy number variations, epigenetic data, may be helpful to further reduce the dimension of this classifier to the gene level. In future, alternative designs may be exploited to represent the module information and do not rely on reference set of tumors.

## Supporting Information

Figure S1 **The percentage of known colorectal cancer (CRC) genes in top 50–500 MDMs inferred from Barrier dataset.** Known CRC genes were collected from the PubGene (A) or OMIM (B). The percentages were compared with those in top differentially expressed genes (t-test genes) with the same number of genes in top ranked N modules, or GO gene sets with the same amount of top ranked N modules.
(TIF)

Figure S2 **The enrichment levels of somatic mutations in top 50–500 most differentially expressed modules(top modules) (orange and dark orange) or most differentially expressed genes by t-test (top t-test genes) (light blue and dark blue) without and with permutated ‘recurrent’ and ‘non-recurrent’ labels, respectively, identified from Barrier dataset.**
(TIF)

Figure S3 **ROC curve of three independent datasets using 34(A: Top500, B: Top1000), 18 (C: Top500; D: Top1000), or 10 (E: Top500; F: Top1000) training samples (orange: German dataset; blue: Barrier dataset; black: GSE5206 dataset).**
(TIF)

Table S1 **The number of genes and maximum p value in Top N modules.**
(DOC)

Table S2 **Member genes of chemokine signaling pathway present in top 100 modules.**
(DOC)

Table S3 **The comparison of classification performance between our module-based method and other recent methods.**
(DOC)
