# Correction: High Accordance in Prognosis Prediction of Colorectal Cancer across Independent Datasets by Multi-Gene Module Expression Profiles

**DOI:** 10.1371/annotation/59a30e2e-76cf-4b51-9489-5bf0c2394afb

**Published:** 2012-09-05

**Authors:** Wenting Li, Rui Wang, Zhangming Yan, Linfu Bai, Zhirong Sun

There was an error in the placement of Figures 2 and 5. The figures were switched. The legends are in the correct locations. 

