# Supplementary figures and images for: High Accordance in Prognosis Prediction of Colorectal Cancer across Independent Datasets by Multi-Gene Module Expression Profiles
Source: PLoS One. 2012 Mar 16;7(3):e33653. doi: 10.1371/journal.pone.0033653 (PMC3306280; doi:10.1371/journal.pone.0033653)

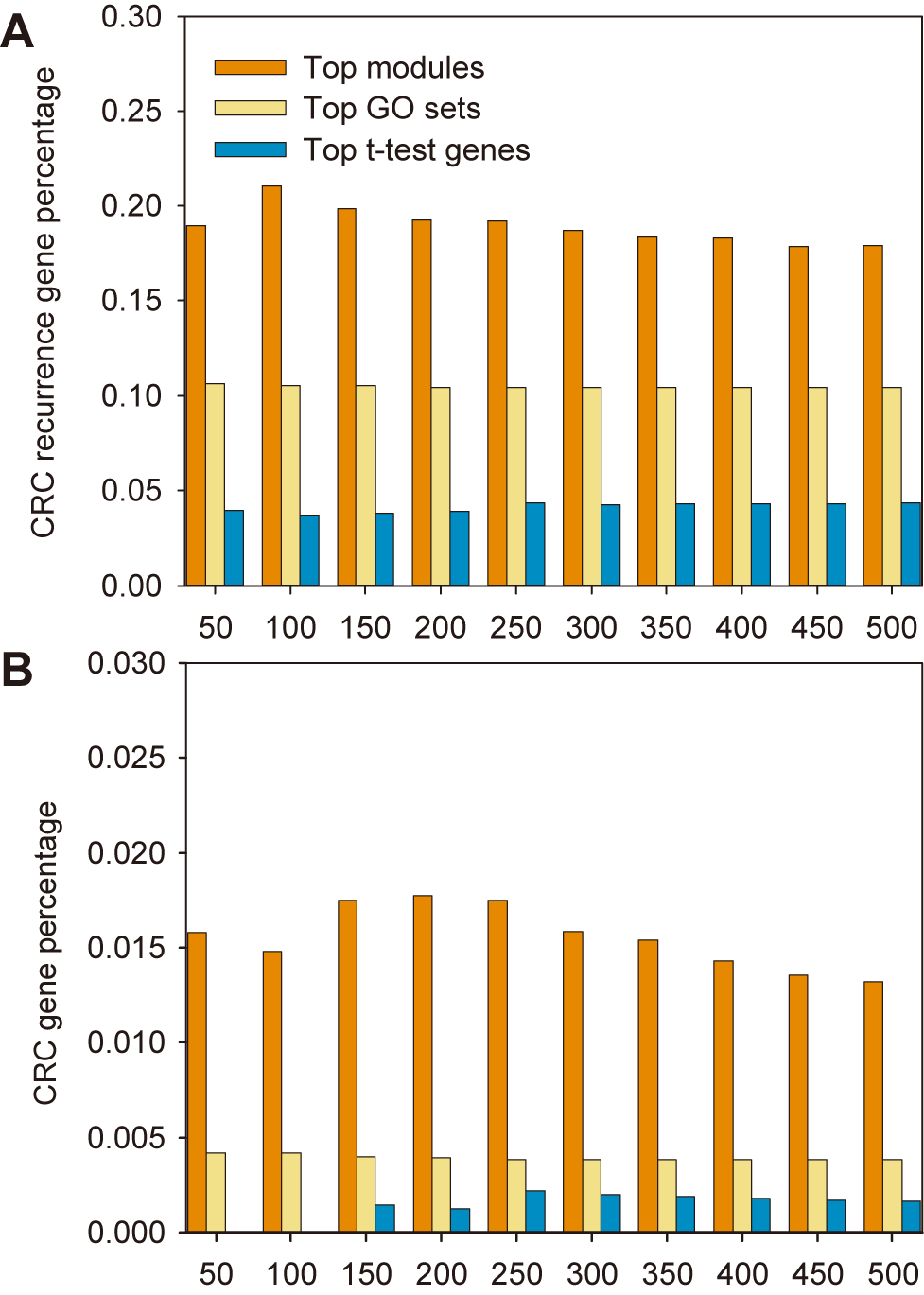

Supplement: Figure S1 — The percentage of known colorectal cancer (CRC) genes in top 50–500 MDMs inferred from Barrier dataset. Known CRC genes were collected from the PubGene (A) or OMIM (B). The percentages were compared with those in top differentially expressed genes (t-test genes) with the same number of genes in top ranked N modules, or GO gene sets with the same amount of top ranked N modules. (TIF) [file pone.0033653.s001.tif]

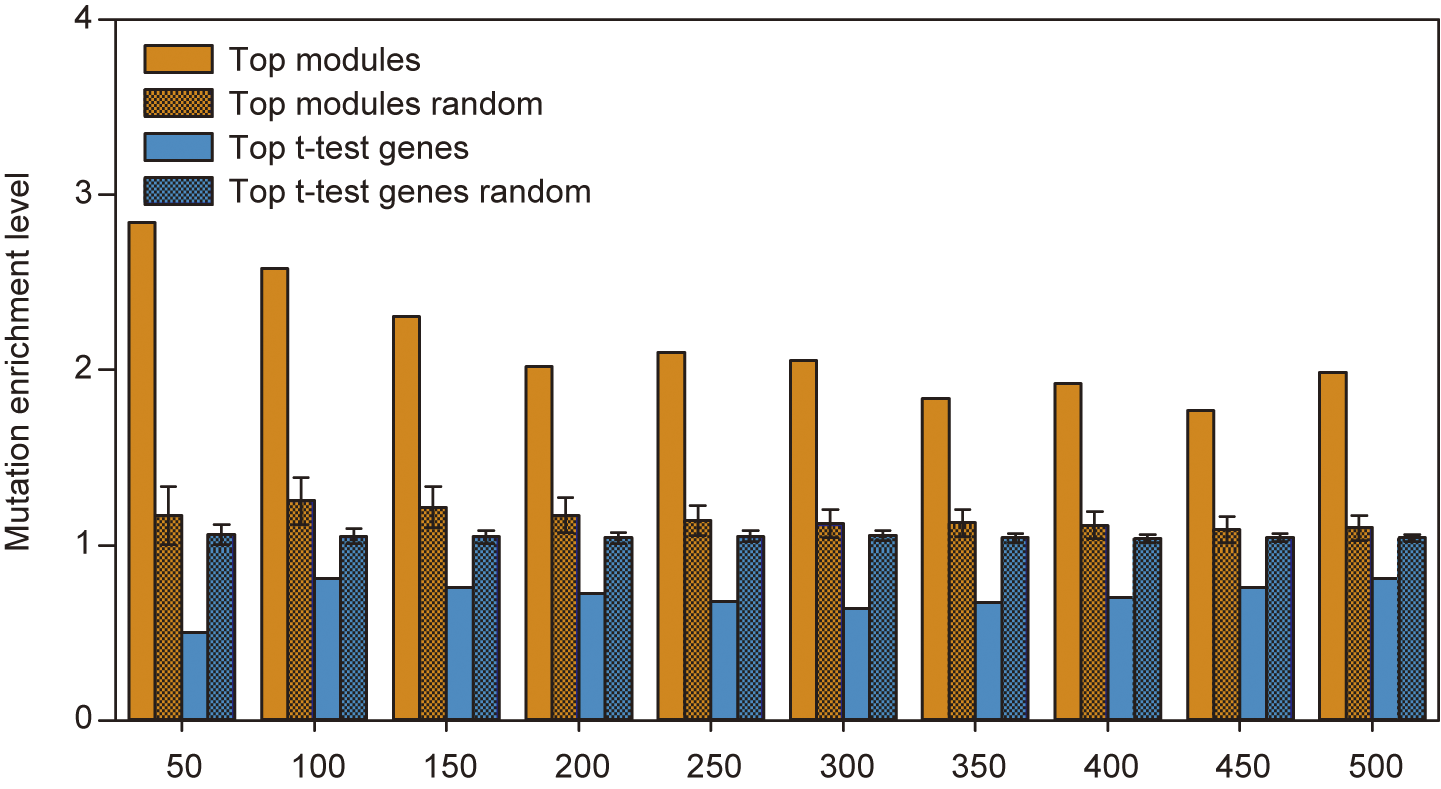

Supplement: Figure S2 — The enrichment levels of somatic mutations in top 50–500 most differentially expressed modules(top modules) (orange and dark orange) or most differentially expressed genes by t-test (top t-test genes) (light blue and dark blue) without and with permutated ‘recurrent’ and ‘non-recurrent’ labels, respectively, identified from Barrier dataset. (TIF) [file pone.0033653.s002.tif]

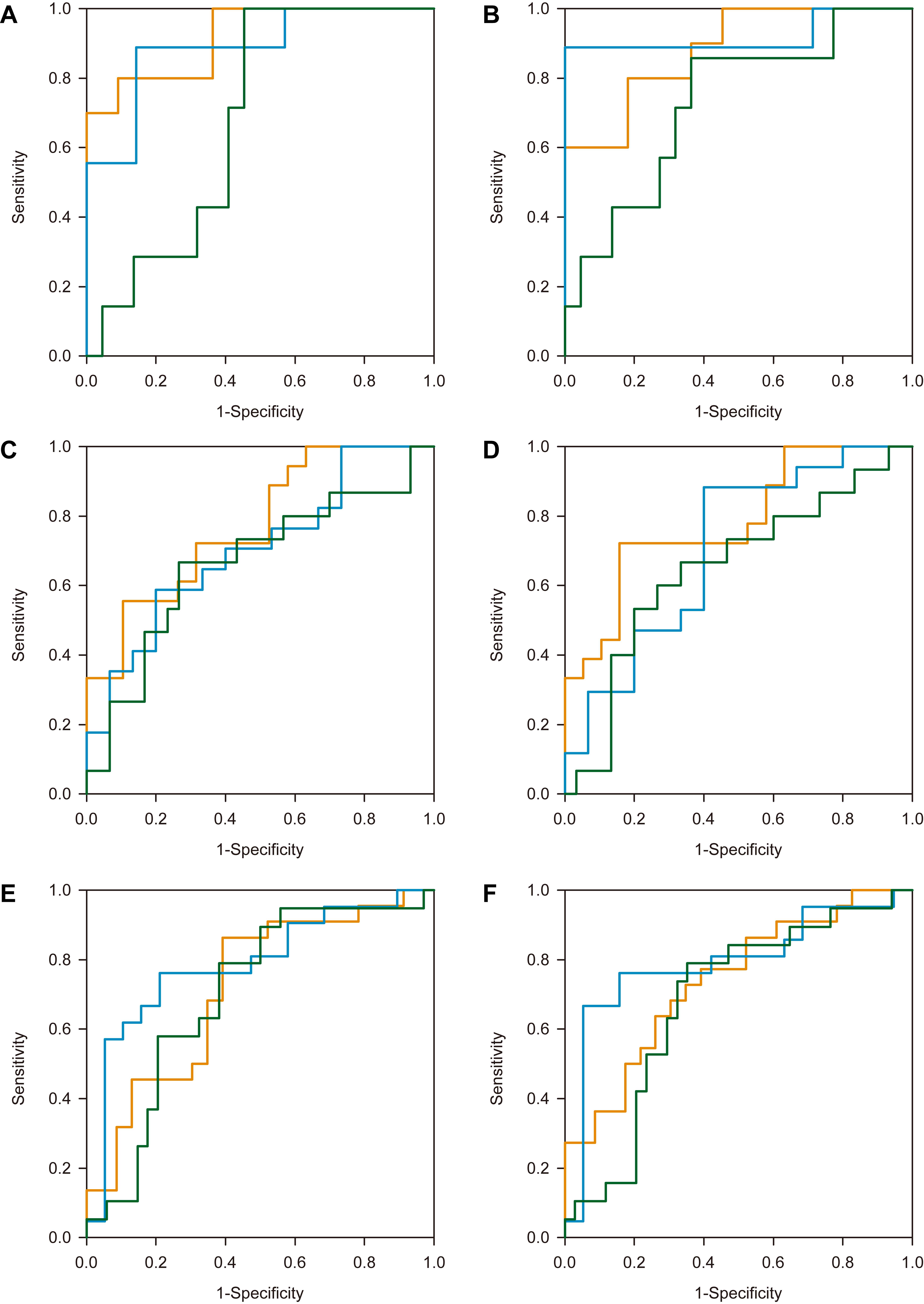

Supplement: Figure S3 — ROC curve of three independent datasets using 34(A: Top500, B: Top1000), 18 (C: Top500; D: Top1000), or 10 (E: Top500; F: Top1000) training samples (orange: German dataset; blue: Barrier dataset; black: GSE5206 dataset). (TIF) [file pone.0033653.s003.tif]
