# Supplementary material for: High Accordance in Prognosis Prediction of Colorectal Cancer across Independent Datasets by Multi-Gene Module Expression Profiles
Source: PLoS One. 2012 Mar 16;7(3):e33653. doi: 10.1371/journal.pone.0033653 (PMC3306280; doi:10.1371/journal.pone.0033653)
Supplement: Table S1 — The number of genes and maximum p value in Top N modules. (DOC) [file pone.0033653.s004.doc]

**Table S1.** The number of genes and maximum p value in Top N modules

|  | German datasets | | Barrier datasets | |
| --- | --- | --- | --- | --- |
| Top N modules | #gene | p | #gene | p |
| 100 | 554 | 3.49E-09 | 541 | 2.69E-06 |
| 200 | 915 | 2.27E-07 | 789 | 1.74E-05 |
| 300 | 1135 | 2.31E-06 | 1009 | 5.92E-05 |
| 400 | 1309 | 1.08E-05 | 1118 | 0.000124 |
| 500 | 1420 | 2.52E-05 | 1211 | 0.000252 |
| 600 | 1550 | 6.11E-05 | 1331 | 0.000426 |
| 700 | 1585 | 8.94E-05 | 1436 | 0.000664 |
| 800 | 1654 | 0.000177 | 1500 | 0.000869 |
| 900 | 1723 | 0.000287 | 1622 | 0.001242 |
| 1000 | 1823 | 0.000555 | 1688 | 0.001695 |
