# Supplementary material for: High Accordance in Prognosis Prediction of Colorectal Cancer across Independent Datasets by Multi-Gene Module Expression Profiles
Source: PLoS One. 2012 Mar 16;7(3):e33653. doi: 10.1371/journal.pone.0033653 (PMC3306280; doi:10.1371/journal.pone.0033653)
Supplement: Table S2 — Member genes of chemokine signaling pathway present in top 100 modules. (DOC) [file pone.0033653.s005.doc]

Table S2. Member genes of chemokine signaling pathway present in top 100 modules

| For German dataset | For Barrier dataset |
| --- | --- |
| STAT1 | STAT2 |
| JAK2 | STAT3 |
| JAK3 | FOXO3 |
| STAT2 | RELA |
| LYN | PTK2B |
| CXCL9 | ROCK2 |
| CXCL11 | NFKB1 |
| CCR3 | PAK1 |
| CXCL10 | LYN |
| CRK | MAPK1 |
| NFKB1 | IKBKB |
| PTK2B | RHOA |
| CXCR4 | HCK |
| PAK1 | RAF1 |
| PRKCZ | AKT1 |
| MAPK1 | CRKL |
| STAT3 | BRAF |
| FOXO3 | GSK3B |
| GSK3B |  |
