# Supplementary material for: High Accordance in Prognosis Prediction of Colorectal Cancer across Independent Datasets by Multi-Gene Module Expression Profiles
Source: PLoS One. 2012 Mar 16;7(3):e33653. doi: 10.1371/journal.pone.0033653 (PMC3306280; doi:10.1371/journal.pone.0033653)
Supplement: Table S3 — The comparison of classification performance between our module-based method and other recent methods. (DOC) [file pone.0033653.s006.doc]

Table S3. The comparison of classification performance between our module-based method and other recent methods.

|  |  | **Accuracy** | | | **Area under curve (AUC)** | | | **Sensitivity** | | | **Specificity** | | |
| --- | --- | --- | --- | --- | --- | --- | --- | --- | --- | --- | --- | --- | --- |
| **datasets** | **methods** | **mean** | **min** | **max** | **mean** | **min** | **max** | **mean** | **min** | **max** | **mean** | **min** | **max** |
| **German** | Top500_34 | 0.78 | 0.71 | 0.86 | 0.80 | 0.75 | 0.91 | 0.80 | 0.70 | 0.90 | 0.76 | 0.64 | 0.91 |
|  | Top1000_34 | 0.75 | 0.71 | 0.81 | 0.79 | 0.73 | 0.88 | 0.78 | 0.70 | 0.90 | 0.73 | 0.64 | 0.82 |
|  | Top500_18 | 0.70 | 0.65 | 0.76 | 0.72 | 0.64 | 0.77 | 0.73 | 0.67 | 0.78 | 0.67 | 0.63 | 0.74 |
|  | Top1000_18 | 0.72 | 0.62 | 0.78 | 0.73 | 0.64 | 0.78 | 0.71 | 0.67 | 0.78 | 0.73 | 0.58 | 0.84 |
|  | Top500_10 | 0.69 | 0.58 | 0.76 | 0.67 | 0.53 | 0.73 | 0.67 | 0.55 | 0.86 | 0.71 | 0.61 | 0.83 |
|  | Top1000_10 | 0.68 | 0.56 | 0.73 | 0.68 | 0.51 | 0.75 | 0.67 | 0.64 | 0.73 | 0.68 | 0.48 | 0.78 |
|  | Top500_LOO | 0.76 | na | na | 0.78 | na | na | 0.65 | na | na | 0.86 | na | na |
|  | Top1000_LOO | 0.76 | na | na | 0.79 | na | na | 0.58 | na | na | 0.93 | na | na |
|  | Lin07_LOO | 0.71 | 0.51 | 0.86 | na | na | na | 0.62 | 0.32 | 0.86 | 0.79 | 0.52 | 0.95 |
|  | Garman08_LOO | 0.69 | na | na | na | na | na | 0.41* | na | na | 0.88* | na | na |
| **Barrier** | Top500_34 | 0.80 | 0.75 | 0.88 | 0.83 | 0.76 | 0.92 | 0.80 | 0.67 | 0.89 | 0.80 | 0.71 | 0.86 |
|  | Top1000_34 | 0.81 | 0.75 | 0.94 | 0.84 | 0.76 | 0.92 | 0.78 | 0.67 | 0.89 | 0.86 | 0.71 | 1.00 |
|  | Top500_18 | 0.64 | 0.56 | 0.69 | 0.64 | 0.56 | 0.69 | 0.61 | 0.59 | 0.65 | 0.68 | 0.47 | 0.80 |
|  | Top1000_18 | 0.66 | 0.53 | 0.75 | 0.64 | 0.52 | 0.70 | 0.62 | 0.53 | 0.88 | 0.71 | 0.53 | 0.87 |
|  | Top500_10 | 0.71 | 0.65 | 0.78 | 0.68 | 0.52 | 0.77 | 0.73 | 0.62 | 0.90 | 0.67 | 0.37 | 0.79 |
|  | Top1000_10 | 0.70 | 0.60 | 0.80 | 0.67 | 0.53 | 0.79 | 0.70 | 0.62 | 0.76 | 0.69 | 0.47 | 0.84 |
|  | Top500_LOO | 0.74 | na | na | 0.76 | na | na | 0.64 | na | na | 0.84 | na | na |
|  | Top1000_LOO | 0.74 | na | na | 0.79 | na | na | 0.72 | na | na | 0.76 | na | na |
|  | Barrier06_LOO | 0.80 | na | na | na | na | na | 0.75 | na | na | 0.85 | na | na |
|  | Barrier06_TS34 | 0.80* | 0.62* | 0.94* | na | na | na | 0.91* | na | na | 0.72* | na | na |
|  | Barrier06_TS18 | 0.74* | 0.59* | 0.85* | na | na | na | 0.80* | na | na | 0.67* | na | na |
|  | Barrier06_TS10 | 0.66* | 0.52* | 0.76* | na | na | na | 0.75* | na | na | 0.57* | na | na |
|  | Barruer06_wang04_TS34 | 0.70* | na | na | na | na | na | na | na | na | na | na | na |
|  | Barrier06_Wang04_TS18 | 0.65* | na | na | na | na | na | na | na | na | na | na | na |
|  | Barrier_Wang04_TS10 | 0.63* | na | na | na | na | na | na | na | na | na | na | na |
|  | Barrier06_Wang04_LOO | 0.67* | na | na | na | na | na | na | na | na | na | na | na |
| **GSE5206** | Top500_34 | 0.70 | 0.66 | 0.76 | 0.66 | 0.64 | 0.68 | 0.74 | 0.57 | 1.00 | 0.68 | 0.55 | 0.82 |
|  | Top1000_34 | 0.68 | 0.59 | 0.76 | 0.66 | 0.63 | 0.72 | 0.74 | 0.57 | 0.86 | 0.66 | 0.50 | 0.82 |
|  | Top500_18 | 0.63 | 0.51 | 0.71 | 0.62 | 0.54 | 0.66 | 0.63 | 0.60 | 0.67 | 0.63 | 0.43 | 0.73 |
|  | Top1000_18 | 0.66 | 0.56 | 0.73 | 0.62 | 0.54 | 0.65 | 0.63 | 0.47 | 0.73 | 0.67 | 0.47 | 0.87 |
|  | Top500_10 | 0.65 | 0.58 | 0.68 | 0.62 | 0.52 | 0.71 | 0.66 | 0.58 | 0.79 | 0.64 | 0.56 | 0.71 |
|  | Top1000_10 | 0.68 | 0.57 | 0.75 | 0.63 | 0.55 | 0.70 | 0.66 | 0.58 | 0.79 | 0.69 | 0.53 | 0.82 |
|  | Top500_LOO | 0.68 | na | na | 0.70 | na | na | 0.61 | na | na | 0.73 | na | na |
|  | Top1000_LOO | 0.65 | na | na | 0.72 | na | na | 0.70 | na | na | 0.63 | na | na |
|  | Garman08_LOO | 0.90 | na | na | 0.94 | na | na | 0.86 | na | na | 0.96 | na | na |

*estimated from graph
